# Rapid resolution of cheilitis granulomatosa with addition of oral metronidazole to a multidrug regimen

**DOI:** 10.1016/j.jdcr.2025.02.048

**Published:** 2025-04-10

**Authors:** Vidya M. Medepalli, Tanner P. Harding, Brittany R. Shectman, Michael J. Bernhardt, Stephen K. Richardson

**Affiliations:** a University of Central Florida HCA Florida Healthcare Graduate Medical Education, Tallahassee, Florida; b NOVA Kiran Patel College of Medicine, Davie, Florida; c Dermatology Associates of Tallahassee, Tallahassee, Florida

**Keywords:** cheilitis granulomatosa, granulomatous cheilitis, metronidazole

## Introduction

Cheilitis granulomatosa (CG), also known as granulomatous cheilitis or cheilitis granulomatosa of Miescher, is a rare disorder primarily affecting young adults with an equal sex distribution.^1^, ^2^, ^3^, ^4^, ^5^, ^6^, ^7^, ^8^, ^9^, ^10^ CG classically presents with recurrent episodes of painless, nonpruritic lip swelling, initially lasting hours to days. As the disease progresses, episodes increase in duration and may lead to cosmetic disfigurement and functional disability. Its etiology remains poorly understood with proposed mechanisms including allergic reactions, infectious reactions, photosensitivity, Th1 immune dysregulation, and a cutaneous manifestation of Crohn’s disease.

Given its rarity, no randomized controlled trials have been conducted to further inform treatment. Historically, CG has been managed with intralesional/intramuscular/topical corticosteroids, clofazimine, dapsone, thalidomide, doxycycline or minocycline, immunosuppressives (eg mycophenolate mofetil, azathioprine), tumor necrosis factor alpha inhibitors, and more recently, the Janus kinase inhibitor, upadacitinib.^1^ Over the past decade, a small but rising number of cases successfully treated with oral metronidazole have been reported, either as monotherapy or part of a multidrug regimen. We describe the case of a 41-year-old male who experienced complete and rapid resolution of refractory CG upon addition of metronidazole to a regimen consisting of doxycycline and pulsed intramuscular corticosteroid.

## Case presentation

**Fig 1:**
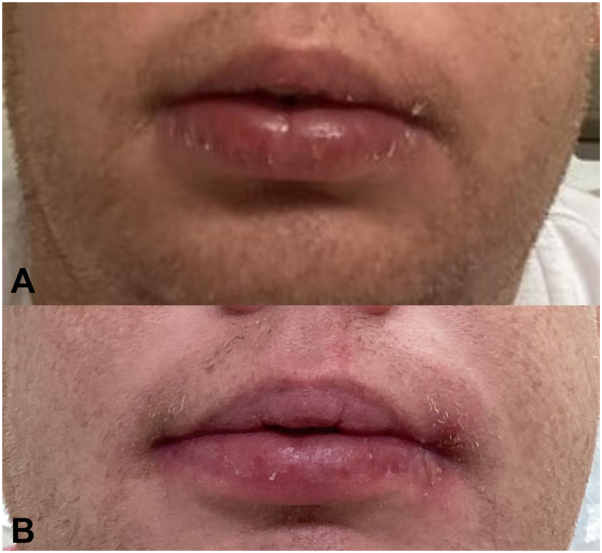
**A,** Week 0, frontal view, demonstrating significant edema and protrusion of the patient’s lower lip. **B,** Week 26, frontal view, demonstrating complete resolution of the lower lip edema and protrusion.

**Fig 2:**
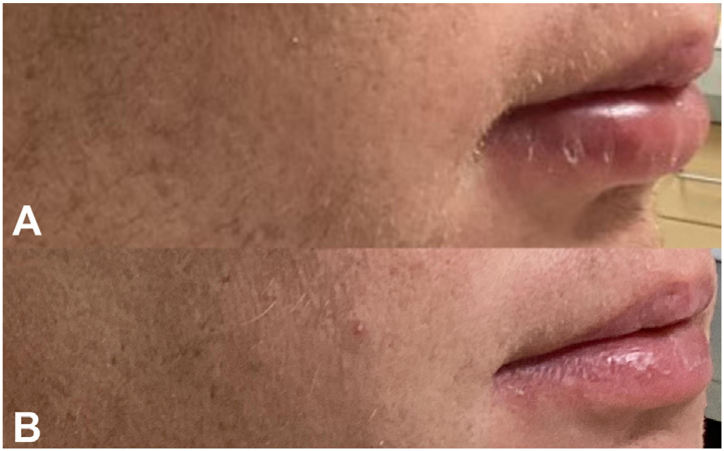
**A,** Week 0, side view, demonstrating significant edema and protrusion of the patient’s lower lip. **B,** Week 26, side view, demonstrating complete resolution of the lower lip edema and protrusion.

**Fig 3:**
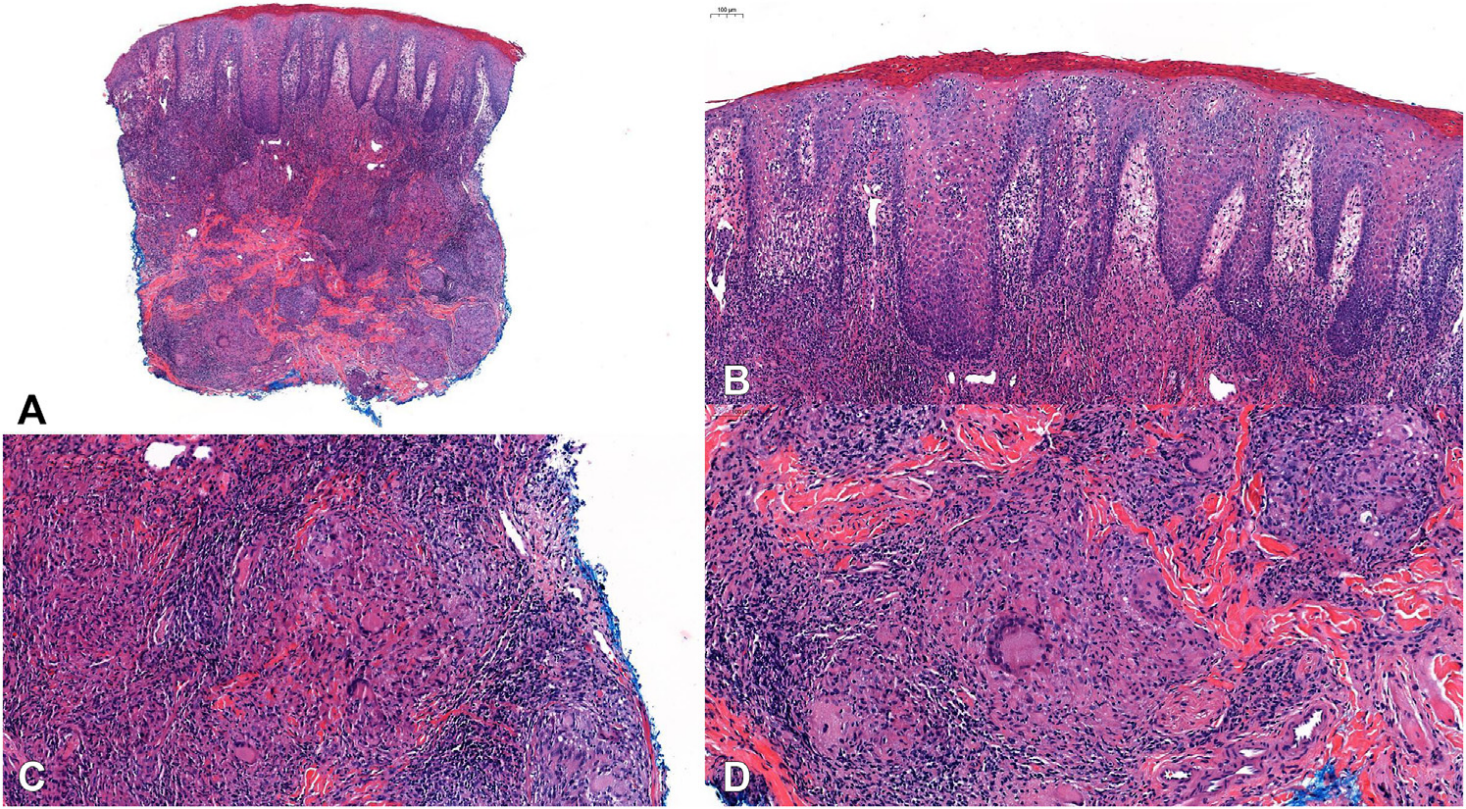
**A,** Hematoxylin-eosin (H&E) stain, original 2.2× magnification, demonstrating a superficial and deep granulomatous dermatitis. **B,** H&E stain, original magnification, 8.2×, demonstrating epidermal acanthosis with epidermal neutrophils and a lichenoid interface dermatitis. **C,** H&E stain, original magnification, 14.1×, demonstrating nodular aggregates of epithelioid histiocytes rimmed by lymphocytes. **D,** H&E stain, original magnification, 15.3×, demonstrating multinucleated giant cells as well as sarcoid-type granulomas.

A 41-year-old male with no significant past medical history presented with a several month history of asymptomatic swelling of his lower lip (Figs 1 and 2). He endorsed functional impairment (drooling and slurred speech) and denied systemic symptoms. His physical examination demonstrated profound protrusion and induration of the lower lip with no associated oral mucosal findings or focal neurologic deficits. A past biopsy performed at another clinic demonstrated histologic findings consistent with CG, including epidermal acanthosis with neutrophils, a lichenoid interface dermatitis, superficial nodular aggregates of epithelioid histiocytes rimmed by lymphocytes, deep sarcoidal type granulomas, and scattered multinucleated giant cells (Fig 3). Sarcoidosis, angioedema, systemic granulomatous disease, mycobacterium tuberculosis infection, and orofacial Crohn’s disease were ruled out by laboratory studies; periodic acid-Schiff, Fite, and acid-fast bacillus stains were negative for microorganisms; angiotensin converting enzyme level, C1 esterase inhibitor level, complement panel, tuberculosis testing, and fecal calprotectin were unremarkable.

Upon presentation to our clinic (week 0), intramuscular triamcinolone acetonide (60 mg) was administered. Additionally, the patient was started on doxycycline 50 mg daily and desonide 0.05% cream twice daily. Despite several months of treatment, the patient noted minimal improvement of his condition. At week 20, another 60 mg dose of intramuscular triamcinolone acetonide was administered and treatment with oral metronidazole (375 mg 3 times daily) was initiated. By week 26, the patient exhibited complete resolution of his lower lip protrusion and symptoms, and his metronidazole dosing was decreased to twice daily. At week 36, his dose was tapered to once daily, and at week 52 it was discontinued in the setting of sustained remission. He remains disease-free 112 weeks after initial evaluation.

## Discussion

An English-language PubMed search of “granulomatous cheilitis” OR “cheilitis granulomatosa” OR “cheilitis granulomatosa of Miescher” AND “metronidazole” yielded 8 reports detailing 11 cases of CG, 10 of which were successfully treated with metronidazole.^2^, ^3^, ^4^, ^5^, ^6^, ^7^, ^8^, ^9^, ^10^ The single reported treatment failure described a patient who exhibited no response (or poor tolerance) to metronidazole but subsequently responded to intralesional corticosteroid.^10^ Of the 10 successfully treated cases (ie, complete clinical resolution of disease), 3 were solely managed with metronidazole,^3^^,^^4^ 3 received metronidazole monotherapy after failing other treatments (ie, ketotifen, intralesional corticosteroids, oral corticosteroids, mesalamine, and/or doxycycline),^5^, ^6^, ^7^ 3 received metronidazole as part of a multidrug regimen at the onset^8^^,^^9^; and 1 patient was started on a combination of metronidazole and minocycline following an inadequate response to systemic corticosteroids.^10^ In the cases described by Dummer et al and Koracin et al, all patients achieved a complete clinical response within 3 weeks of starting metronidazole. Our case offers additional evidence in support of oral metronidazole as an effective treatment for refractory CG. Through the induction of DNA strand breakage, metronidazole interferes with protein synthesis and is commonly prescribed for protozoal and anaerobic bacterial infections. It also possesses anti-inflammatory and immunomodulatory properties, likely accounting for its therapeutic benefit in the setting of CG among other inflammatory disorders.

Topical formulations of metronidazole are readily available and US Food and Drug Administration approved for the treatment of inflammatory rosacea. Although clinically distinct, the granulomatous variant of rosacea and CG exhibit histologic similarities, particularly the presence of granulomas. Current literature does not report the use of topical metronidazole for CG; however, given its efficacy for the treatment of granulomatous rosacea, it conceptually may offer potential benefit for CG patients, such as sustaining clinical remission. Although our patient’s clinical response cannot be solely attributed to oral metronidazole, the resolution of his disease was hastened by the addition of metronidazole to his treatment regimen.

## Conclusion

CG is a rare disease that has historically been difficult to manage. A growing number of reported cases support the therapeutic benefit of oral metronidazole as a monotherapy or part of a multidrug treatment regimen. Particularly noteworthy is the rapid resolution of disease noted in our patient. The Janus kinase inhibitor, upadacitinib, has also shown great promise as a treatment option, with complete responses achieved within 3-5 months.^1^ As more cases are reported, larger studies may inform future treatment decisions. Additionally, the efficacy of topical metronidazole in the setting of granulomatous rosacea implies a shared pathophysiologic mechanism with CG that may be worthy of further investigation, as it could impact how we classify these disorders. In the interim, clinicians should be cognizant of the potential benefit of oral metronidazole as a first-line therapy for CG given both its well-recognized safety profile and reports of rapid efficacy.

## Conflicts of interest

None disclosed.
